# MTSplice predicts effects of genetic variants on tissue-specific splicing

**DOI:** 10.1186/s13059-021-02273-7

**Published:** 2021-03-31

**Authors:** Jun Cheng, Muhammed Hasan Çelik, Anshul Kundaje, Julien Gagneur

**Affiliations:** 1 Department of Informatics, Technical University of Munich, Boltzmannstraße, Garching, 85748 Germany; 2 Department of Computer Science, Stanford University, Stanford, CA USA; 3 Department of Genetics, Stanford University, Stanford, CA USA; 4 Institute of Computational Biology, Helmholtz Zentrum München, Neuherberg, Germany; 5 Institute of Human Genetics, Klinikum rechts der Isar, Technical University of Munich, Munich, Germany

## Abstract

We develop the free and open-source model Multi-tissue Splicing (MTSplice) to predict the effects of genetic variants on splicing of cassette exons in 56 human tissues. MTSplice combines MMSplice, which models constitutive regulatory sequences, with a new neural network that models tissue-specific regulatory sequences. MTSplice outperforms MMSplice on predicting tissue-specific variations associated with genetic variants in most tissues of the GTEx dataset, with largest improvements on brain tissues. Furthermore, MTSplice predicts that autism-associated de novo mutations are enriched for variants affecting splicing specifically in the brain. We foresee that MTSplice will aid interpreting variants associated with tissue-specific disorders.

**Supplementary Information:**

The online version contains supplementary material available at (10.1186/s13059-021-02273-7).

## Introduction

Splicing is a fundamental biological process in which introns are cut out from precursor RNAs and exons are joined together. Alternative splicing refers to alternative usage of exons. It is estimated that approximately 95% of human multi-exon genes undergo alternative splicing [1]. Exon skipping (of so-called cassette exons) is the most common alternative splicing pattern [2]. Skipping level of an exon is commonly quantified with the percent spliced-in (PSI or *Ψ*) [3]. Percent spliced-in can be estimated from RNA-sequencing (RNA-Seq) data as the number of split RNA-Seq reads supporting the inclusion of the exon divided by the total number of split reads supporting the skipping or the inclusion of the exon. Splicing is a complex process which involves regulation by sequence elements in the exons and flanking introns [4, 5]. Moreover, alternative splicing is often tissue-specific [2, 3, 6, 7]. This means that certain splicing isoforms are only present in certain tissues or that the relative abundances of splice isoforms differ across tissues. Alternative splicing plays an important role in tissue development and shaping tissue identity [8, 9]. Analyzing the protein-coding roles of tissue-specific exons revealed their critical role in rewiring protein interaction networks in different tissues [10]. Tissue-specific splicing patterns are associated with short RNA motifs [2, 11–14]. These short RNA motifs encode tissue-specific splicing regulatory elements, typically intronic or exonic binding sites for splicing factors with a tissue-specific activity. Mammalian tissue-specific splicing factors include Nova1, Nova2, PTB/nPTB, and RBFOX1 for nervous tissues, and MBNL1 for muscles, among others. For a review, see Chen and Manley [15].

Splicing defects account for an important fraction of the genetic basis of human diseases [16–18]. Some of these splicing defects are specific to disease-relevant tissues. For instance, individuals affected by autism spectrum disorder (ASD) frequently present mis-splicing of brain-specific exons [19–21] as well as an enrichment of de novo mutations in brain-specific exons [22]. Hence, computational tools that can predict the tissue-specific effects of genetic variants on splicing would be relevant for understanding the genetic basis of tissue-specific diseases such as ASD.

Many computational tools have been developed to predict splice sites or splicing strength from sequence [23–33]. However, tools are lacking for predicting tissue-specific effects of human genetic variants on splicing. Barash et al. developed the first sequence-based model predicting tissue-specific splicing in mouse cells [34]. The model integrates regulatory sequence elements to qualitatively predict whether the inclusion of a cassette exon increases, decreases, or remains at a similar level from one tissue to another tissue. This model was further improved to predict directional changes between tissues along with discretized *Ψ* categories (low, medium, and high) within a tissue by using a Bayesian neural network with hidden variables [35]. In a subsequent study, a similar Bayesian neural network (SPANR) was trained on human data [29]. However, SPANR was evaluated only for predicting the largest effect across all investigated tissues. Hence, the performance of SPANR on any given tissue is unclear. Moreover, the publicly available SPANR does not allow performing tissue-specific predictions.

We previously developed MMSplice, a neural network with a modular design that predicts the effect of variants on splicing [30, 31]. Unlike SPANR, which has been trained on natural endogenous genomic sequence, MMSplice leverages perturbation data from a recently published massively parallel reporter assay [28]. MMSplice outperformed SPANR and many other splicing predictors in predicting *Ψ* variations associated with naturally occurring genetic variants as well as effects of variants on percent spliced-in measured on reporter assays [30, 36]. MMSplice models the odds ratio of a cassette exon to be spliced-in when comparing an alternative sequence to a reference sequence. The predicted odds ratios are the same for all tissues because MMSplice has been trained in a tissue-agnostic fashion and therefore does not capture effects of variants affecting tissue-specific regulatory elements.

Deep learning models of tissue-specific regulatory elements have been developed for other biological processes. These models include DeepSEA for chromatin-profiles [37], Basset for DNase I hypersensitivity [38], ExPecto for tissue-specific gene expression [39], FactorNet for transcription factor binding [40], and ChromDragoNN for chromatin accessibility [41]. A common denominator of these models is that they are trained by multi-task learning, i.e., the models make joint predictions for all tissues or cell types using a common set of underlying predictive features. This strategy allows models to efficiently pool information about regulatory elements that are shared across cell types or tissues.

Here, we developed MTSplice (Multi-tissue Splicing), a model that predicts tissue-specific splicing effects of human genetic variants. MTSplice adjusts the MMSplice predictions with the predictions of TSplice (Tissue-specific Splicing), a novel deep neural network predicting tissue-specific variations of *Ψ* from sequence which we trained on 56 human tissues using multi-task learning. Performance of MTSplice is demonstrated by predicting tissue-specific variations of *Ψ* associated with naturally occurring genetic variants of the GTEx dataset as well as investigating brain-specific splicing effect predictions for autism-associated variants. MTSplice is open-source and freely available at the model repository Kipoi [42].

## Results

### Tissue-specific alternatively spliced exons

To train a tissue-specific model of splicing, we considered the alternative splicing catalog of the transcriptome ASCOT [43]. Because the ASCOT annotation and quantification pipeline is annotation-free, it also covers non-annotated exons. Altogether, ASCOT provides *Ψ* values for 61,823 cassette exons across 56 tissues including 53 tissues from the GTEx dataset [44] and additional RNA-Seq data from peripheral retina. Of note, these tissue-specific values are flagged as missing when the corresponding gene is not expressed [43].

**Fig. 1:**
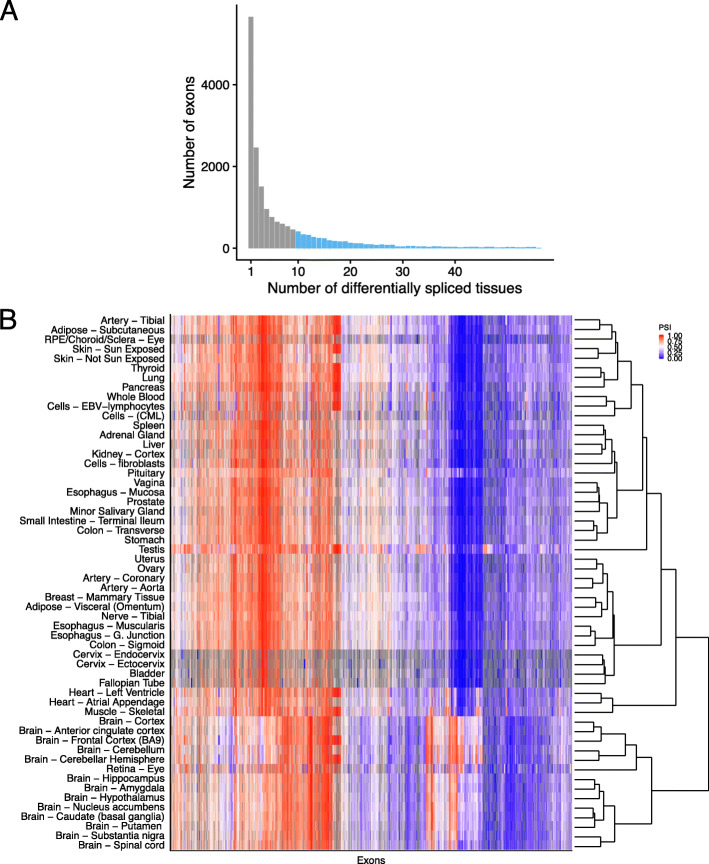
Differential splicing of alternatively spliced exons across tissues. **a** Histogram of the number of tissues with differential splicing (*Ψ* deviating by at least 10% from the exon-average *Ψ*). Overall, 4398 exons (light blue) are differentially spliced in at least 10 tissues. **b**. Heatmap of *Ψ* for the 4398 exons that are differentially spliced in at least 10 tissues with exons (columns) and tissues (rows) sorted by hierarchical clustering. *Ψ* is color-coded by a gradient from blue (0) to red (1) via white (0.5). Gray entries are missing values and occur in tissues for which the corresponding gene is not expressed. Hierarchical clustering was applied after imputing missing values with row means

Overall, *Ψ* of 17,991 exons (29%) of the ASCOT dataset deviate by at least 10% in at least one tissue from its exon-specific average across tissues. These deviations from the exon-specific average *Ψ* by 10% often occurred in a single tissue (5658 exons, 31%) and in at least 10 tissues for 4398 exons (25%, Fig. 1a). We investigated co-variations between tissues using these 4398 exons (Fig. 1b). This revealed that samples from the central nervous system (brain, spinal cord, and retina) have very distinct splicing patterns compared to other tissues, in agreement with previous reports [24]. Moreover, skeletal muscle and the two heart tissues (left ventrial and artial appendage) also clustered together with shared splicing patterns. Altogether, this analysis indicates that the ASCOT dataset provides thousands of tissue-specific splicing events that could be used to train a sequence-based predictive model. Also, the ASCOT dataset provides the possibility for a multi-task model to exploit shared splicing regulation of tissues of the central nervous system and, to a lower extent, between skeletal muscle and cardiac tissues.

### Differential splicing associated with genetic variants show little tissue-specific variations

**Fig. 2:**
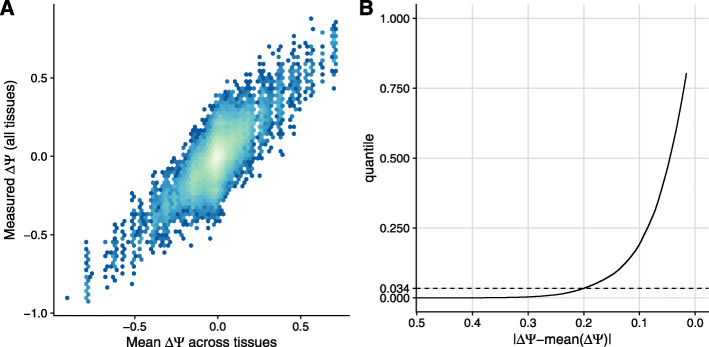
Tissue-specific variations of differential splicing associated with genetic variants in the GTEx dataset. **a** Tissue-specific differential *Ψ* associated with a genetic variant (*y*-axis) against differential *Ψ* associated with a genetic variant averaged across tissues (*x*-axis). Effects were estimated using homozygous donors (**b**). Proportion of data points shown in **a** (*y*-axis) for every cutoff on the deviation from the averaged *Ψ* across tissues (*x*-axis, decreasing)

The ASCOT dataset consists of data aggregated per tissue. In principle, the genetic variations between donors of the original GTEx dataset provide further information that a sequence-based model could exploit. We therefore next asked how much genetic variation among individuals in GTEx associated with tissue-specific splicing variations. To this end, we computed *ΔΨ*, the difference between *Ψ* averaged across individuals homozygous for the alternative allele and *Ψ* averaged across individuals homozygous for the reference allele for exons with a single variant within the exon body and 300 nucleotides flanking the exon either side (“Materials and methods” section). We estimated *Ψ* using the software for estimating splice isoform abundances MISO [45], which only takes annotated and alternatively spliced exons into account. Over all these 1767 single-nucleotide variants, little tissue-specific deviation of *ΔΨ* compared to its average across tissues was observed (Fig. 2a). Specifically, less than 1476 instances (3.4% of exon-variant-tissue pairs) of tissue-specific *ΔΨ* deviated by 20% from the tissue-averaged *ΔΨ* (Fig. 2b). This observation is consistent with the fact that only a limited fraction (between 7 and 21%) of splicing QTLs are tissue-specific [46]. Since GTEx samples are derived from healthy donors, this observation, however, does not rule out the possibility that some disease-causing variants do alter splicing in a tissue-specific way. Due to the small amount of tissue-specific splicing variation associated with genetic variants in GTEx, we decided to train a sequence-based model solely based on the variations between exons using the ASCOT aggregated data and to keep the genetic variations between donors of the GTEx dataset to independently assess the model afterward.

### TSplice predicts tissue-specific *Ψ*

**Fig. 3:**
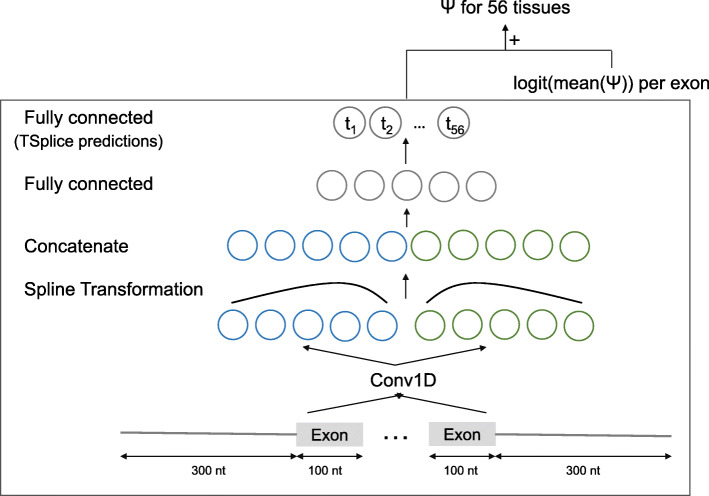
Model architecture to predict tissue-specific percent spliced-in. The model TSplice consists of one convolution layer with 64 length-9 filters capturing sequence elements from one-hot encoded input sequences. This is followed by two spline transformation layers modulating the effect of sequence elements depending on their position relative to the acceptor splice sites (leftmost layer) and the donor (rightmost layer). The outputs of the two spline transformation layers are concatenated, and global average pooling is applied along the sequence dimension. This is then followed by feeding two consecutive fully connected layers. The last fully connected layer outputs a 56-dimension vector which are the predicted log odds ratios of tissue-specific *Ψ* versus tissue-averaged *Ψ* for the 56 tissues of the ASCOT dataset. Natural scale tissue-specific *Ψ* are obtained by adding predicted odds ratios with measured tissue-averaged *Ψ* on the logit scale. Batch normalization was used after all layers with trainable parameters except the last fully connected layer. In total, the model has 8024 trainable parameters

We next developed a neural network, TSplice, to predict tissue-specific *Ψ* values from sequence and tissue-averaged *Ψ* (“Materials and methods” section). TSplice considers the 300 nt flanking either side of the exon and the first and last 100 nt of the exon body. TSplice is a convolutional neural network (Fig. 3) in which positional effects of sequence elements relative to splice sites are modeled using spline transformations [47]. TSplice was trained on the ASCOT dataset using all chromosomes except for chromosome 2, 3, and 5. We report our model prediction performances on these held-out chromosomes.

**Fig. 4:**
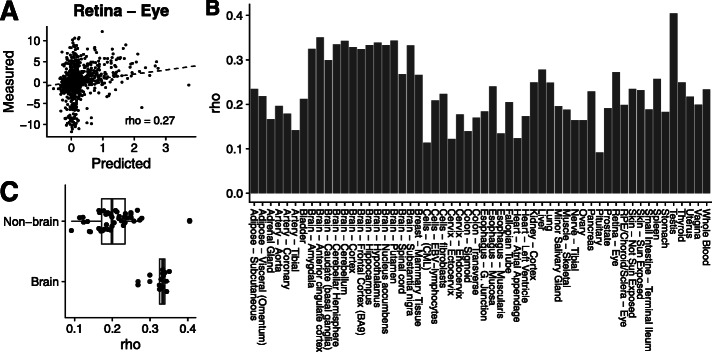
Evaluating TSplice on predicting tissue-associated differential splicing. **a** Predicted versus measured tissue-associated differential splicing for the retina eye tissue, representative of the typical performance of our model. **b** Spearman correlation between predicted and measured tissue-associated differential splicing for all tissues. **c** Distribution of Spearman correlations between predicted and measured tissue-associated differential splicing for brain tissues and non-brain tissues

The performance of TSplice was first assessed on test data by comparing the observed against the predicted log odds ratios of tissue-specific *Ψ* for 1621 exons (“variable exons”) with *Ψ* deviating from the tissue-averaged *Ψ* by at least 0.2 in at least one tissue and for which the gene is expressed in at least 10 tissues (Fig. 4a for the retina eye as an example, Spearman *ρ*=0.27). The predictions positively correlated with the measurements in all tissues and showed a median Spearman correlation of 0.22 (Fig. 4b, Additional file 1: Fig. S1). The performance was higher for tissues of the central nervous system (Fig. 4c), possibly because central nervous system tissues harbor similar splicing patterns and because they are well represented in the ASCOT dataset.

**Fig. 5:**
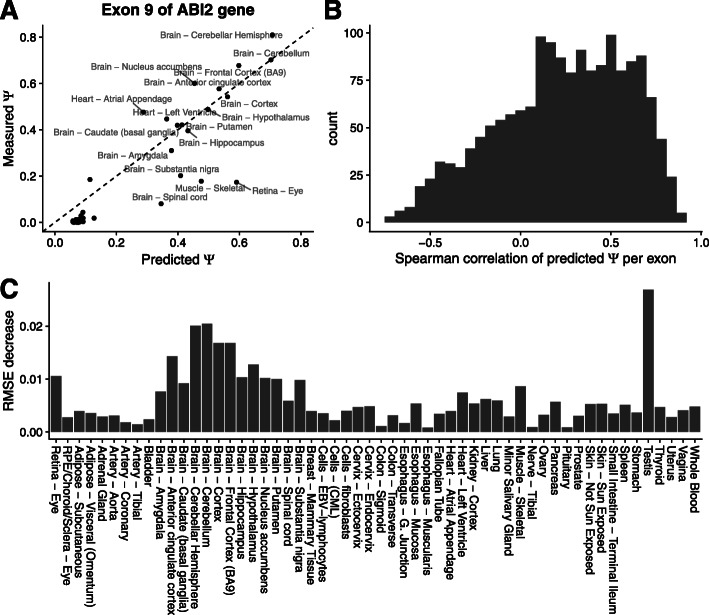
Evaluating TSplice on predicting tissue-specific *Ψ*. **a**. Predicted (*x*-axis) versus measured (*y*-axis) *Ψ* for the 9^th^ exon of gene ABI2 across 56 tissues. **b**. Histogram of the Spearman correlation of the predicted versus measured *Ψ* for 1621 test exons across 56 tissues. **c** Root-mean-square error decreases between the TSplice model and the baseline model (predicted with the mean *Ψ* across tissues)

We had first assessed log odds ratio predictions because these are the actual quantities the model was trained for. However, percent spliced-ins on the natural scale often matter more for biological and medical applications. We hence next evaluated how well TSplice performs on predicting tissue-specific *Ψ* on test exons. A successful example is the 9^th^ exon of the gene ABI2, which is included in brain, heart, muscle, and retina tissues and for which TSplice predicts well the order of the tissues (Fig. 5a, Spearman *ρ*=0.8) and the absolute values of tissue-specific *Ψ* per-tissue (root-mean-square error, short RMSE, 0.11). For the majority of the variable exons (73.9%, 1198 out of 1621), TSplice ranked tissue-specific *Ψ* in the right direction (median *ρ*=0.25, Fig. 5b). We benchmarked TSplice against a *L*_2_ regularized linear model based on known splice-regulating motifs and splice site sequences (“Materials and methods” section). Although the performance (Spearman correlation of predicted versus measured *Ψ*_e,t_) of the alternative model correlates (*R*=0.373) with TSplice across tissues, TSplice outperforms the alternative model for all 56 tissues (Additional file 1: Fig. S2A). Furthermore, when evaluated per exon, TSplice had higher Spearman correlation than the alternative model for 63.5% of the exons (*P*<2.2×10^−16^, paired Wilcoxon test, Additional file 1: Fig. S2B).

Visualization of the positional weights learned by the splines of TSplice showed that some filters were important for the 5^′^ half of the model, others for the 3^′^ half, while about a third of them were important for both halves. Moreover, positional effects were particularly marked near the splice sites (Additional file 1: Fig. S3). Visualizing the model gradient with respect to the input sequence indicated that the model activates at sequences matching binding site motifs of the splicing factors PTBP1/2, NOVA1/2, and MBNL1 (Additional file 1: Fig. S4 for examples, “Materials and methods” section). To study the role of these motifs systematically, we next generated in silico mutated sequences by injecting the consensus sequences of these splicing factor binding sites at various positions along 1000 randomly selected sequences of the test set. We then computed the TSplice score difference between each mutated sequence and its original counterpart. TSplice predicted that cassette exons with a NOVA1/2 binding site consensus sequence in the upstream intron are less spliced-in (i.e., more skipped) in the brain compared to other tissues on average (Additional file 1: Fig. S5). Since the RNA-binding protein NOVA1 is neuron-specifically expressed, these TSplice predictions are consistent with a NOVA1 repressive role when binding in the upstream intron [11]. Moreover, exons with an MBNL1 binding site consensus sequence in the upstream intron are predicted to be more spliced-in in the brain and muscle than in other tissues on average (Additional file 1: Fig. S5). This is consistent with the repressive role of MBNL1 when binding to upstream introns and with MBNL1 being less expressed in the brain and muscle than in other tissues [48]. The interpretation of the effects of PTBP1/2 binding site consensus sequence is more complex since it is recognized by two competing factors with anti-correlated expression during neuronal differentiation [49].

Altogether, these results show that TSplice captured sequence features predictive of *Ψ* changes across tissues.

### Tissue-specific variant effect prediction

**Fig. 6:**
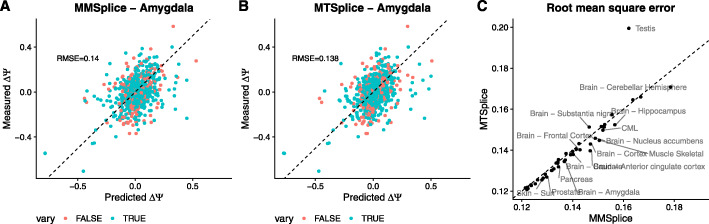
Comparing MMSplice and MTSplice on predicting variant-associated differential splicing. **a**, **b** Predicted (*x*-axis) versus measured (*y*-axis) *ΔΨ* in amygdala between alternative and reference alleles for variants with between-tissue splicing variation (orange) and other variants (cyan) for MMSplice (**a**) and MTSplice (**b**). **c** Root-mean-square error of MTSplice predictions (*y*-axis) against MMSplice predictions (*x*-axis) for exons with between-tissue splicing variations (the cyan dots in **a**). Each dot represents one of the 51 GTEx tissues with at least 10 measured variant effects. MTSplice improves for 39 tissues, yet mildly, over MMSplice. Tissues for which the RMSE differences larger than 0.002 are labeled with text

We next considered combining MMSplice, which models tissue-independent effects together with TSplice, which models differential effects between tissues, to predict the effects associated with genetic variants for any GTEx tissue (“Materials and methods” section). We name this combined model MTSplice. For amygdala, taken as a representative tissue, the MTSplice predictions correlate well (*ρ*=0.42, Fig. 6a) with differences of *Ψ* observed between homozygous donors (“Materials and methods” section). This is consistent with the observation that most variants have similar effects across tissues. Nevertheless, MTSplice further improved the prediction accuracy when evaluated on 1030 variants with *ΔΨ* varying by at least 0.2 in at least one tissue (RMSE = 0.140 for MMSplice alone, RMSE = 0.138 for MTSplice, RMSE = 0.141 versus 0.139 when evaluated on all variant, Fig. 6). When evaluated on the 51 tissues with at least 10 measured variant effects, MTSplice outperformed MMSplice for 39 out of 51 tissues in terms of root-mean-square error (*P*=1.76×10^−5^, paired Wilcoxon test, Fig. 6c). Notably, MTSplice outperformed MMSplice in 10 out of 12 brain tissues (Additional file 1: Fig. S6A). Although the improvement of MTSplice over MMSplice are significant, the relative decrease of RMSE remains modest. The relative increases were more pronounced when restricting the analysis to those measurements harboring large tissue-specific effects (Additional file 1: Fig. S6B).

### MTSplice predicts brain-specific signals for autism patients

**Fig. 7:**
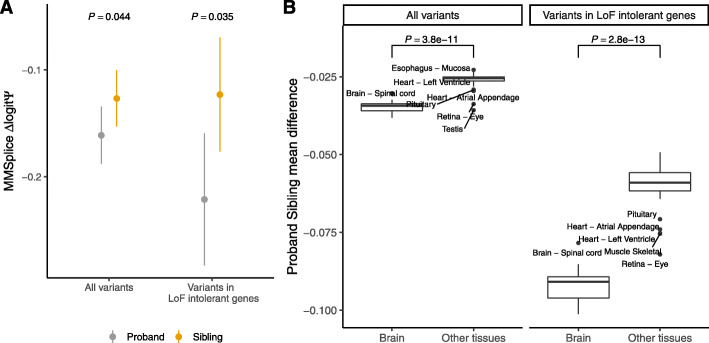
Brain-specific mutational burden on splicing in ASD. **a** Tissue-agnostic variant effect prediction with MMSplice. Splice-region de novo mutations (*n* = 3884, “Materials and methods” section) of the proband group (gray) have significantly lower predicted *Δ*logit*Ψ* according to MMSplice compared to those of the unaffected sibling group (orange). The effect size is larger for variants in LoF-intolerant genes (*n* = 1081). Shown are the means and standard 95% confidence intervals. *P* values from one-sided Wilcoxon test. **b**. Tissue-specific variant effect prediction with MTSplice. Distribution of effect size (difference of average *Δ*logit*Ψ* for proband versus control siblings de novo mutations) for brain tissues (right boxes) and other tissues (left boxes), and for all de novo mutations (left panel) or de novo mutations in LoF-intolerant genes (right panel) with MTSplice. Individual tissue plots are shown Additional file 1: Fig. S8. The predicted effect sizes are more pronounced for brain tissues

To assess the potential of MTSplice on scoring tissue-specific disease variants, we considered de novo mutations that were reported for 1790 autism spectrum disorder (ASD) simplex families from the Simons Simplex Collection [50–54] and as provided by Zhou et al. [55]. The data consists of 127,140 de novo mutations, with 65,147 from the proband group and 61,993 from the unaffected siblings. Of those, we further considered the 3884 mutations lying in exons or in their 300-nt flanking intronic regions and predicted with MMSplice with a *Δ*logit(*Ψ*) magnitude greater than 0.05. Overall, MMSplice predicted that variants of the proband group would disrupt splicing more strongly than variants of the control siblings (negative MMSplice scores, Fig. 7a, *P*=0.042, Wilcoxon rank-sum test). The effect was even stronger for the 1081 loss-of-function (LoF) intolerant genes (Fig. 7a, *P*=0.0035, Wilcoxon rank-sum test, “Materials and methods” section). This result is consistent with the report that LoF-intolerant genes are vulnerable to noncoding disruptive mutations in ASD [55] and points to an important contribution of splicing.

We then asked whether MTSplice was able to identify tissue-specific effects of ASD-associated de novo mutations. Consistent with the MMSplice results, the de novo mutations of the proband group were predicted by MTSplice to more severely disrupt splicing than the de novo mutations of the control group for all tissues (Fig. 7b). The effect size was larger for the brain tissues (Fig. 7b). Since autism is a neurological disorder, these results indicate that MTSplice may be used to prioritize variants that could play a tissue-specific pathogenic role. Besides the brain tissues, the tissues with most pronounced differences were the retina, which is also part of the central nervous systems and muscle, which has been associated with autism as well [56]. These differences were further amplified when restricting the analysis to the de novo mutations in LoF-intolerant genes (Fig. 7b).

We next asked whether MTSplice could capture tissue-specific disease signal that would otherwise be missed by MMSplice. Among the mutations predicted to have small effects by MMSplice (*Δ*logit(*Ψ*) magnitude smaller than 0.05), the predicted effect for the proband group is indeed no severe than the control sibling group (Additional file 1: Fig. S7A). Moreover, when considering the tissue-specific effect predicted by MTSplice, the de novo mutations from the proband group were predicted to disrupt more severely splicing in the frontal cortex (Additional file 1: Fig. S7A, *P*=0.036, one-sided Wilcoxon test). Furthermore, these ASD-associated signals were generally found in the brain, heart, muscle, and retina but not in other tissues (Additional file 1: Fig. S7B). This shows that MTSplice is able to capture tissue-specific disease signals that would have been missed by MMSplice.

Altogether, these analyses demonstrate the value of MTSplice on predicting tissue-specific effects of potentially disease-causing mutations.

## Discussion

We introduced the model MTSplice which quantitatively predicts effects of human genetic variants on RNA splicing in 56 tissues. MTSplice has two components. One component, MMSplice, models constitutive splicing regulatory sequences. The other component, TSplice, models tissue-specific splicing regulatory sequences. The combined model MTSplice outperforms MMSplice on predicting tissue-specific variations in percent spliced-in associated with naturally occurring genetic variants in most tissues of the GTEx dataset. Applying MTSplice to de novo mutations from autism spectrum disorder simplex families [55], we found a significantly higher burden for the proband group compared to the control siblings, particularly in brain tissues. These results suggest that MTSplice could be applied for scoring variants with a tissue-specific pathogenic role.

Various lines of evidence indicated that our model performed best for brain tissues. This may reflect the fact that brain tissues are well represented in GTEx but also that tissue-specific alternative splicing is particularly strong in brain tissues, giving more useful sequences to train on. Supportive of this, model interpretation revealed that sequence elements known to be bound by brain-specific splicing factors contributed to TSplice predictions. For other tissues, the improvements were more moderate yet consistent. One exceptional tissue is the testis, for which MMSplice still has a substantially better RMSE than MTSplice for variant effect predictions. We could not rationalize that observation. Perhaps, this could be due to the unique transcriptional state of the testis which may affect splicing in a way that the model failed to learn [57].

The TSplice component was trained from tissue-specific alternative splicing observed in the ASCOT dataset. This approach has two main limitations. First, only less than ten thousand exons show tissue-specific alternative splicing in the ASCOT dataset. This amount of data prohibits training of more complex models. In comparison, MMSplice was trained using over 2 million sequences of a massively parallel reporter assay and over half a million naturally occurring splice sites. To overcome this limitation, one could leverage complementary data notably tissue-specific expression of splicing-related RNA-binding proteins (RBPs) combined with transcriptome-wide RBP binding profiles [58]. One example of a transfer learning approach in this context is given by Jha et al. [59], who showed the benefits of integrating CLIP-Seq data to predict splicing. The second limitation is that the ASCOT dataset is an observational dataset. Models trained from observational data with genomic sequences may learn sequence features that are correlative but not causal, preventing the models from correctly predicting the effect of genetic variants. This could lead to limited predictive performances of our current model.

One approach to overcome the issue of observational data is to perform massively parallel reporter assays (MPRA) for different cell types. MPRA for human splicing have been performed in HEK293 cells [26, 28, 60–62], K562 cells [63, 64], HepG2 cells [63], and HELA and MCF7 cells [65]. These data provide powerful resources to train complex models on splicing, but tissue and cell-type diversity is still lacking. Tissue-specific MPRA data would also be of prime importance for benchmarking models. Here we had to rely on naturally occurring variants in GTEx for benchmarking. Tissue-specific alteration of splicing can be the outcome of genetic variation affecting either (i) constitutive splicing regulatory elements of tissue-specific exons or (ii) tissue-specific splicing regulatory elements. Very few GTEx variants were from the latter class. Hence, the mean square error differences in GTEx between MTSplice and MMSplice could only be very mild. Previous two-cell-line splicing MPRA experiment did not find tissue-specific variant effects between K562 and HepG2 cells [63], maybe also because the variants tested were selected randomly. A designed MPRA, however, could specifically engineer variations of tissue-specific splicing regulatory elements by using prior knowledge in order to more deeply probe the effect of variants on tissue-specific splicing regulation. The generation of large-scale tissue or cell-type-specific perturbation data could therefore be instrumental for probing tissue-specific regulatory elements and could yield more sensitive benchmarks of predictive models. Other than improvement on the training data side, future models might be able design better architecture and data augmentation techniques to further improve performance. Finally, because the approach to predict tissue-specific variant effects by combining MMSplice with a tissue splicing-level prediction model is general, any model that outputs tissue-specific logit(*Ψ*_e,t_) could substitute to TSplice and be combined with MMSplice to predict tissue-specific variant effect on splicing.

## Materials and methods

### Dataset

We split the 61,823 cassette exons from ASCOT into a training, a validation, and a test set. The training set consisted of 38,028 exons from chromosome 4, 6, 8, 10-23, and the sex chromosomes. The 11,955 exons from chromosome 1, 7, and 9 were used as the validation set, and the remaining 11,840 exons were used as the test set (chromosomes 2, 3, and 5). Models are evaluated based on their performance on the test set.

### Variant effect estimation

To compute variant effect, we first computed *Ψ* with MISO for all annotated alternatively spliced exons (MISO annotation v2.0, http://genes.mit.edu/burgelab/miso/annotations/ver2/miso_annotations_hg19_v2.zip) in all GTEx RNA-Seq samples. This led to *Ψ* estimates for 4686 samples from 53 tissues. Second, for each exon, we estimated variant effects using only those samples with a single variant within the exon body and 300 nt flanking of the exon. Third, we estimated the effect associated with the variants as the difference between *Ψ* averaged across samples homozygous for the alternative allele and *Ψ* averaged across samples homozygous for the reference allele. We required at least 2 samples in each of these two groups. For simplicity, we did not consider heterozygous samples for estimating the effects because *Ψ* of heterozygous samples is confounded by allele-specific RNA expression. Also, we did not consider indels.

### The TSplice model

We denote *Ψ*_*e*,*t*_ the percent spliced-in value of the cassette exon *e* in tissue *t*. The goal of the multi-tissue splicing model is to predict tissue-specific *Ψ*_*e*,*t*_ from the nucleotide sequence of the given exon S_*e*_. We train the tissue-specific splicing model with multi-task learning, where each task corresponds to a tissue. The model has two input branches. The first input branch consists of the sequence 300 nt upstream of the acceptor and 100 nt downstream of the acceptor (Fig. 3). In a symmetric fashion, the second input branch consists of the sequence from the donor side, with 100 nt upstream of the donor and 300 nt downstream of the donor. All input sequences are one-hot encoded. The input layer is followed by a 1D convolution layer with 64 filters of length 9. Parameters of the convolution layer are shared by the two input branches, based on the assumption that many sequence motifs are presented both upstream and downstream of the exons. To model the positional effects of splicing motifs, spline transformations [47] are fitted for each of the convolution filters to weight the convolution activations based on the relative input position to donor and acceptor sites. The spline transformations are fitted differently for the two input branches to account for potential different positional effects of the upstream and downstream introns. The weighted activations are then concatenated along the sequence dimension. Two fully connected layers are followed after the concatenated outputs. The last fully connected layer output number of predictions equals the number of tissues (*T*), corresponding to predictions for each tissue. These are the predictions of the TSplice model mentioned in the manuscript. During training, logit of the mean *Ψ* per exon ($\text {logit}(\overline {\Psi }_{e})$) was added to these prediction outputs followed by a sigmoid function. This encourages the model to learn sequence features associated with differential splicing across tissues.

Formally, for each exon, TSplice predicts for each tissue its *Ψ*_e,t_ deviation from the mean $\overline {\Psi }_{\mathrm {e}}$ across tissues on logit level. Specifically, we define the tissue-associated differential splicing as *Δ*_tissue_logit(*Ψ*_*e*,*t*_)

1 $$  \Delta_{\text{tissue}}\text{logit}(\Psi_{e, t}) := \text{logit}(\Psi_{e,t}) - \text{logit}(\overline{\Psi}_{e})  $$

as the logit *Ψ* deviation for tissue *t* and exon *e* from the logit of $\overline {\Psi }_{e} := \frac {1}{T}\sum \limits _{t=1}^{T}\Psi _{e,t}$, the mean *Ψ* across tissues.

For exon *e* with input sequence S_*e*_, TSplice predicts the target in $\mathbb {R}^{T}$: TSplice(S_*e*_):=(*Δ*_tissue_logit(*Ψ*_*e*,1_),...,*Δ*_tissue_logit(*Ψ*_*e*,*T*_)) corresponding to *T* tissues.

The tissue-specific *Ψ*_*e*,*t*_ can be predicted with TSplice and the given $\text {logit}(\overline {\Psi }_{e})$ computed from the data as:

2 $$ \hat\Psi_{e,t} = \sigma \left(\text{TSplice}(\mathrm{S}_{e})_{t} + \text{logit}(\overline{\Psi}_{e})\right)  $$

where TSplice(S_*e*_)_*t*_ is the TSplice predicted *Δ*_tissue_logit(*Ψ*_*e*,*t*_), and *σ* is the sigmoid function: $\sigma (x) = \frac {1}{1+e^{-x}}$. Note that in Eq. 1 and elsewhere the average was computed before and not after logit-transformation because it gave more robust results.

### Model training and selection

The model was implemented with keras (version 2.2.4). The Kullback–Leibler (KL) divergence between the predicted and measured *Ψ* distribution was used as the loss function (Eq. 3), by considering the percent spliced-in as the probability of the cassette exon to be included in any given transcript.

3 $$  \text{Loss} = \frac{1}{T \cdot E} \sum_{t=1}^{T} \sum_{e=1}^{E} \gamma_{e,t} \left(\Psi_{e,t}\log(\frac{\Psi_{e,t}}{\hat\Psi_{e,t}}) + (1-\Psi_{e,t})\log(\frac{1-\Psi_{e,t}}{1-\hat\Psi_{e,t}}) \right),  $$

where

4 $$ \gamma_{e,t} = \left\{\begin{array}{ll} 1, & \text{if} \; \Psi_{e,t} \; \text{observed} \\ 0, & \text{otherwise} \end{array}\right.  $$

Missing values, which typically correspond to tissues in which the gene is not expressed, were masked out in the loss function. *Ψ* values were clipped to be between [10^−5^,1−10^−5^]. Adam optimizer [66] with default parameters was used to optimize the model. Network weights were initialized with the He Normal initialization [67]. Hyperparameter search was performed with hyperopt [68] with the Tree Parzen Estimators method along with the package kopt (https://github.com/Avsecz/kopt). Hyperparameters were selected based on the loss on the validation set.

After finding the best hyperparameter combination, 20 models were trained with the best hyperparameters but different random initialization. A forward model selection strategy was used to select a set of models whose average predictions gives the smallest loss on the validation set. To this end, models were first sorted based on their performance on the validation set. Next, models were successively added to an ensemble model, defined as the average over the selected models, until the validation set performance no longer improved. This procedure yielded an ensemble model composed of 8 individual models. TSplice predictions are made by this ensemble model.

### Alternative tissue-specific *Ψ*_*e*,*t*_ prediction model

The following model was considered as alternative model to predict tissue-specific $\hat \Psi _{e,t}$: For each tissue *t*, we train a *L*_2_ regularized linear regression model (ridge regression):

5 $$  \text{logit}(\Psi_{\text{e,t}}) = \beta_{0,t} + \sum_{i} \beta_{i,t} X_{i} + \text{logit}(\Psi_{\text{e,average}}) + \epsilon_{t},  $$

where *X*_*i*_ are the sequence features shared for all tissues. Sequence features from five regions were considered separately: upstream intron, exon, downstream intron, donor and acceptor. For upstream and downstream introns, we considered TCAT (NOVA1/2), TGCATG (RBFOX1), GCTTGC (MBNLl), and all 6-mers with A/T (PTBP1/2) [69–72]. For exons, we considered NOVA1/2, RBFOX1 and MBNL1 motifs and 2272 exonic splicing regulators identify by [26]. For all motifs, *X*_*i*_ are the vectors of counts of motif instances in the considered region. Acceptor and donor splice site contexts were also considered as for MaxEntScan [24]. On the acceptor side, 20 nt in the intron and 3 nt in the exon were considered. On the donor side, 6 nt in the intron and 3 nt in the exon were considered.

The alternative model was trained on the training and evaluation set, with hyperparameters chosen by cross-validation on the training and evaluation set. The performances were assessed for the same test than TSplice.

### Splicing motif analysis

Splicing motif logos were visualized with contribution scores computed with gradient times input. Examples of motifs were manually selected. Systematic motif discovery with contribution scores was unsuccessful. Motif instances were picked by manually inspecting the activation scores on the input sequences. Motif binding protein was determined by searching motif instances in the ATtRACT database [73]. Position weight matrix (PWM) for PTBP1/2 and MBNL1 were downloaded from RNACompete [74]. PWM for NOVA1/2 which is missing from RNACompete was downloaded from the RBPDB [75]. *P* values for motif matching were computed with TOMTOM [76] and motif database from RNACompete [74].

To visualize the predicted effects of motifs across tissues, sequence motifs were inserted into the native sequences of 1000 randomly selected exons. Scores were computed by subtracting the TSplice predictions with the inserted motifs and without the motifs. Scores were computed for all tissues and all positions. The heatmaps visualize the scores averaged across 1000 exons by tissues and positions.

### Tissue-specific variant effect prediction

Tissue-specific variant effect *ΔΨ*_*e*,*t*_ is predicted as follows (we considered in this study only homozygous cases as described in the “Variant effect estimation” section in the “Materials and methods” section):

6 $$ \Delta\Psi_{\text{e,t}} = \Psi_{\text{e,t}}^{\text{alt}} - \Psi_{\text{e,t}}^{\text{ref}}  $$

where $\Psi _{\text {e,t}}^{\text {ref}}$ is the measured *Ψ* for exon *e* and tissue *t* with the reference sequence, and $\Psi _{\text {e,t}}^{\text {alt}}$ is the tissue-specific *Ψ* with the alternative sequence. We model the logit level of $\Psi _{e,t}^{\text {alt}}$ with the following linear model:

7 $$  \text{logit}(\Psi_{\text{e,t}}^{\text{alt}}) = \beta_{0} + \beta_{\text{tissue}} + \beta_{\text{alt}} + \beta_{\text{alt}\times\text{tissue}} + \epsilon,  $$

where *β*_0_ is intercept, *β*_tissue_ is the tissue effect, *β*_alt_ is the effect of the variant on an average tissue, *β*_alt×tissue_ is the interaction term which we model the interaction of the variant effect and the given tissue. We model each of the terms as follows:

8 $$  \begin{aligned} \beta_{0} &= \text{logit}(\Psi_{\text{e,average}}^{\text{ref}}) \\ \beta_{\text{tissue}} &= \text{TSplice}(\mathrm{S}_{\text{ref}},\text{tissue}) \\ \beta_{\text{alt}} &= \text{MMSplice}(\mathrm{S}_{\text{ref}},\mathrm{S}_{\text{alt}}) \\ \beta_{\text{alt}\times\text{tissue}} &= \text{TSplice}(\mathrm{S}_{\text{alt}},\text{tissue}) - \text{TSplice}(\mathrm{S}_{\text{ref}},\text{tissue}) \end{aligned}  $$

When we plug Eq. 8 into Eq. 7, we obtain the MTSplice model which combines MMSplice and TSplice to model tissue-specific variant effect:

9 $$  \text{logit}(\Psi_{\text{e,t}}^{\text{alt}}) = \text{logit}(\Psi_{\text{e,average}}^{\text{ref}}) + \text{MMSplice}(\mathrm{S}_{\text{ref}},\mathrm{S}_{\text{alt}}) + \text{TSplice}(\mathrm{S}_{\text{alt}},\text{tissue}) + \epsilon  $$

Finally, the tissue-specific *ΔΨ*_e,t_ is predicted as follows:

10 $$  \begin{aligned} \Delta\Psi_{\text{e,t}} = & \sigma\Big(\text{logit}(\Psi_{\text{e,average}}^{\text{ref}}) + \text{MMSplice}(\mathrm{S}_{\text{ref}},\mathrm{S}_{\text{alt}}) + \text{TSplice}(\mathrm{S}_{\text{alt}},\text{tissue}) \Big) \\ & - \Psi_{\text{e,t}}^{\text{ref}} \end{aligned}  $$

While the prediction with MMSplice is as follows:

11 $$ \begin{aligned} \Delta\Psi_{\text{e,t}} = \sigma\Big(\text{logit}(\Psi_{\text{e,average}}^{\text{ref}}) + \text{MMSplice}(\mathrm{S}_{\text{ref}},\mathrm{S}_{\text{alt}}) \Big) - \Psi_{\text{e,t}}^{\text{ref}} \end{aligned}  $$

### Benchmark variant effect prediction on GTEx

On the benchmark of tissue-specific variant effect prediction, we further applied four filters. First, we selected variants that have |*ΔΨ*_e,t_−*ΔΨ*_e,average_|>0.2 in at least one tissue. Second, *ΔΨ* can be computed for at least 3 tissues. Third, we only considered tissues with more than 10 variants satisfying the above criteria. Altogether, these filters led to 1030 variant-exon pairs and 51 tissues used for benchmarking tissue-specific variant effect predictions.

To benchmark variants only in tissues where their effects are tissue-specific, we selected for each variant only the tissues where |*ΔΨ*_e,t_−*ΔΨ*_e,average_|>0.2. Only tissues with at least 10 valid variants are considered. In total, 48 tissues were considered for this analysis.

### Autism variants

The processed de novo mutations were downloaded from the link provided by Zhou et al. [55] (https://hb.flatironinstitute.org/asdbrowser/). The original whole genome sequencing data were accessed through the Simons Foundation Autism Research Initiative (SFARI) [50–54]. The data provides 127,140 single-nucleotide variants (SNVs) from non-repeat-region. The variants were derived from 7097 whole genomes from the Simons Simplex Collection (SSC) cohort, which consists of whole-genome sequencing data from 1790 families (with probands and matched unaffected siblings).

To predict variant effect on splicing, variants were mapped to exons if they are within the annotated (ensembl gene annotation v75) exon body or within 300 nt flanking. If a variant was mapped to multiple exons, the largest effect size was reported as the effect of the variant. A total of 13,415 variants were mapped to known exons and therefore were predicted by our models. Among those variants, 3884 have predicted |*Δ*logit(*Ψ*)|>0.05. We classified the variants into loss-of-function (LoF) group and loss tolerant group based on the loss-of-function observed/expected (oe) upper bound fraction (LOEUF) scores [77]. We used the suggested cutoff of 0.35 on the upper bound of the oe confidence interval to group the variants.

## Supplementary Information

**Additional file 1** Supplementary Figures S1-S8.

**Additional file 2** Review history.

## Data Availability

MTSplice is integrated with MMSplice and is available from https://github.com/gagneurlab/MMSplice_MTSplice[78] under the MIT license, and also deployed at kipoi http://kipoi.org/models/MMSplice/mtsplice/[79]. The source code version used in the manuscript is available from 10.5281/zenodo.4255942[80]. The software comes along with reference *Ψ* tables. For novel cassette exons, users can provide their own *Ψ* table. Analysis code is available from https://gitlab.cmm.in.tum.de/gagneurlab/mtsplice[81] under the MIT license. ASCOT data is available from http://ascot.cs.jhu.edu/.
