# Publisher Correction: MTSplice predicts effects of genetic variants on tissue-specific splicing

**DOI:** 10.1186/s13059-021-02338-7

**Published:** 2021-04-15

**Authors:** Jun Cheng, Muhammed Hasan Çelik, Anshul Kundaje, Julien Gagneur

**Affiliations:** 1 Department of Informatics, Technical University of Munich, Boltzmannstraße, 85748 Garching, Germany; 2 Department of Computer Science, Stanford University, Stanford, CA USA; 3 Department of Genetics, Stanford University, Stanford, CA USA; 4 Institute of Computational Biology, Helmholtz Zentrum München, Neuherberg, Germany; 5 Institute of Human G enetics, Klinikum rechts der Isar, Technical University of Munich, Munich, Germany

**Correction to: Genome Biol 22, 94 (2021)**

**https://doi.org/10.1186/s13059-021-02273-7**

Following publication of the original paper [1], it was noticed that a typesetting error occurred. Julien Gagneur was mistakenly not indicated as a corresponding author. This has been corrected and the original article [1] has been updated.
