# Supplementary material for: MTSplice predicts effects of genetic variants on tissue-specific splicing
Source: Genome Biol. 2021 Mar 31;22:94. doi: 10.1186/s13059-021-02273-7 (PMC8011109; doi:10.1186/s13059-021-02273-7)
Supplement: Supplementary file 1 — Additional file 1 Supplementary Figures S1-S8. [file 13059_2021_2273_MOESM1_ESM.pdf]

MTSplice predicts effects of genetic variants on tissue-specific splicing

1 Supplementary Figures

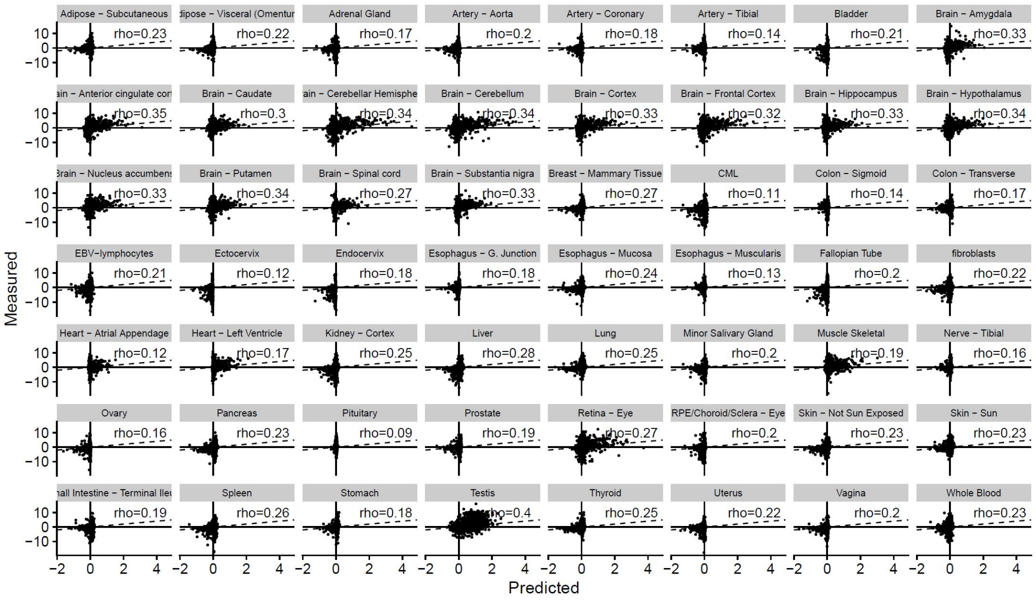

Fig. S1: Predicted (x-axis) versus measured (y-axis) tissue-associated differential splicing. The dashed line indicates the x=y line.

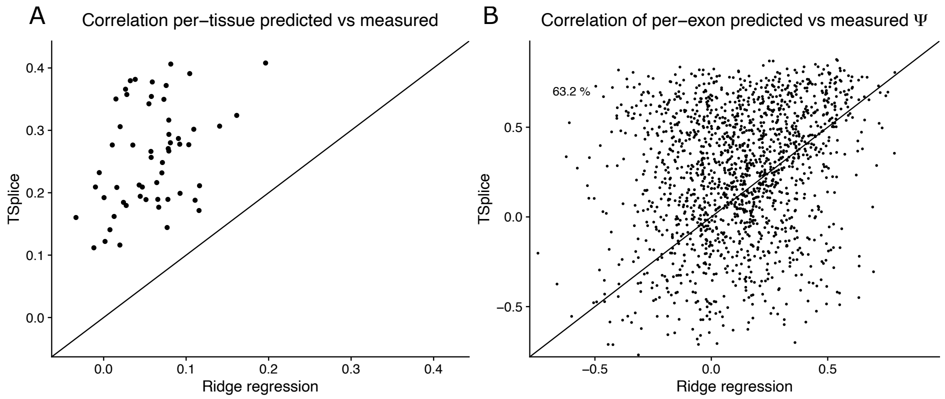

Fig. S2: **(A)**. Spearman correlation of prediction against measured $\Psi_{e,t}$ across 1,621 test exons for the 56 tissues for TSplice (y-axis) against the ridge regression baseline model (x-axis). **(B)** Spearman correlation of prediction against measured $\Psi_{e,t}$ across tissues for 1,621 test exons for TSplice (y-axis) against the ridge regression baseline model (x-axis). The 1,621 exons in the test set are selected for i) being variable (Ψ ($|\Psi_{e,t} - \Psi_{e,\text{average}}| > 0.2$) in at least one tissue and ii) being expressed in at least 10 tissues.

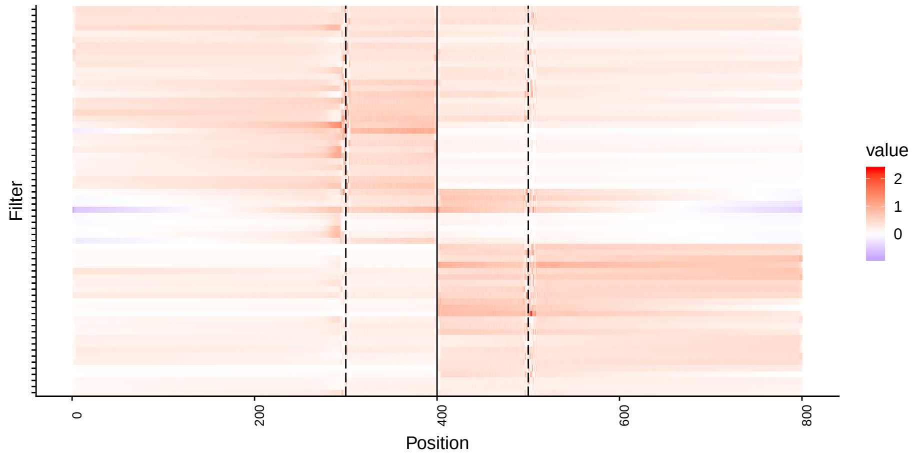

Fig. S3: Average activation map of the convolution layer weighted by the spline transformation layer. Dashed lines represent exon-intron boundary.

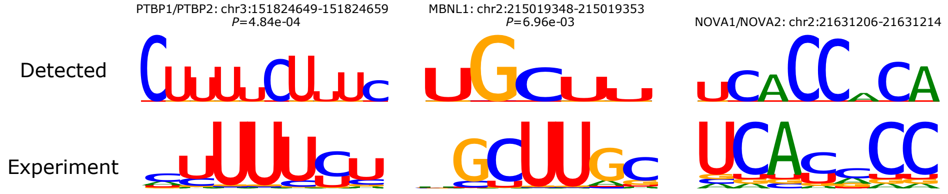

Fig. S4: Examples of motif instances detected by TSplice model. Model detected motifs (*Detected*) were shown along with known experimental derived motifs (*Experiment*).

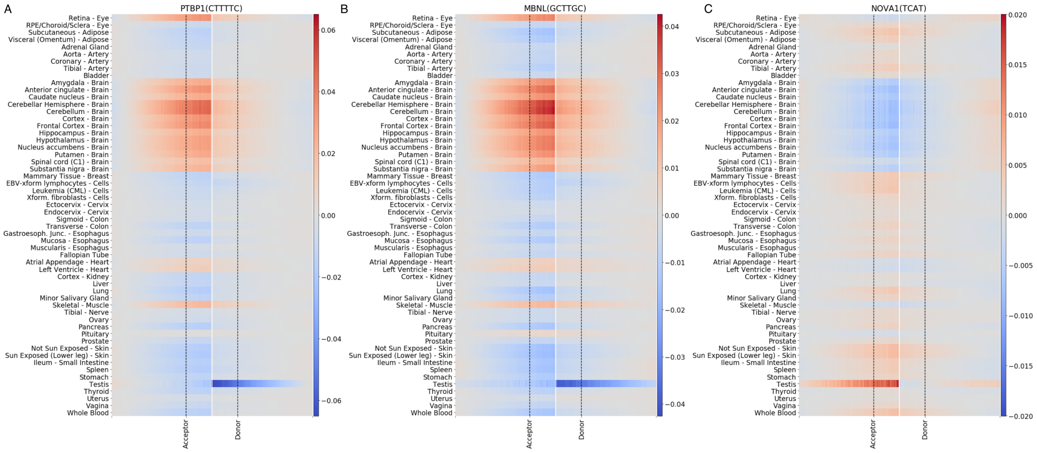

Fig. S5: Predicted motif effects by TSplice on various positions (x-axis) across 56 tissues (y-axis). Effects computed by subtracting TSplice predictions with and without inserted motifs. Values across positions and tissues averaged across 1,000 random selected exons in the test set. Dashed lines indicate exon boundaries.

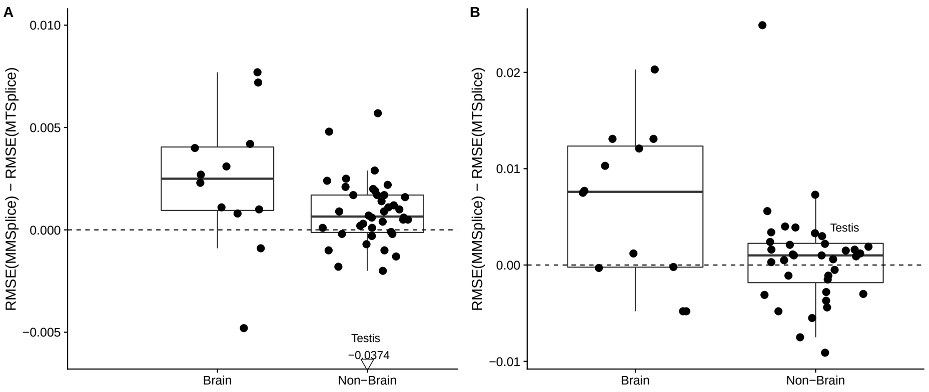

Fig. S6: Comparing MTSplice and MMSplice (RMSE reduction of MTSplice compared to MMSplice, y-axis) stratified by brain and non-brain tissues (x-axis) for the 1,030 variants with a tissue-specific effect ($|\Delta\Psi_{e,t} - \Delta\Psi_{e,average}| > 0.2$ in at least one tissue).**(A)** using data for all tissues and **(B)** restricting to variant-tissue pairs for which $|\Delta\Psi_{e,t} - \Delta\Psi_{e,average}| > 0.2$.

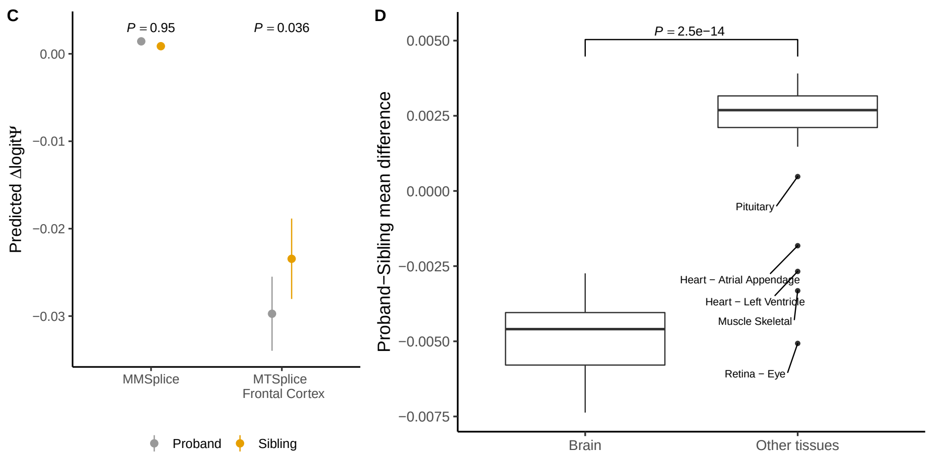

Fig. S7: Predictions of MMSplice and MTSplice on variants with small MMSplice scores ($|\Delta\,\text{logit}(\Psi)| < 0.05$). **(A)** Means (dot) and standard 95% confidence intervals (line) of predicted variant effects ($\Delta\,\text{logit}\,\Psi$, y-axis) for proband (gray) and the unaffected sibling group (orange) with MMSplice (left) or MTSplice in frontal cortex as a particular example (right). The P-values are computed with the one-sided Wilcoxon test. **(B)** Distribution of effect size (difference of average $\Delta\,\text{logit}\,\Psi$ for proband versus control siblings *de novo* mutations) for brain tissues (left box) and other tissues (right box) with MTSplice.

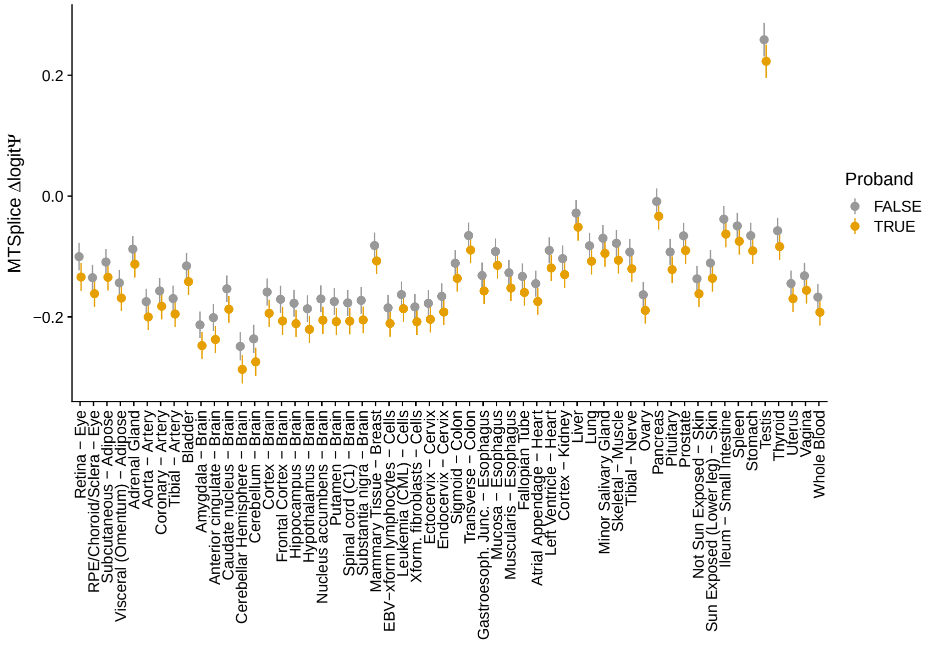

Fig. S8: Tissue-specific variant-effect prediction with MTSplice. MTSplice predicted variant-effect ($\Delta\,\mathrm{logit}\,\Psi$, y-axis) for proband (gray) and the unaffected sibling group (orange) across 56 tissues. The means and standard 95% confidence intervals were shown.
