# Supplementary material for: MTSplice predicts effects of genetic variants on tissue-specific splicing
Source: Genome Biol. 2021 Mar 31;22:94. doi: 10.1186/s13059-021-02273-7 (PMC8011109; doi:10.1186/s13059-021-02273-7)
Supplement: Supplementary file 2 — Additional file 2 Review history. [file 13059_2021_2273_MOESM2_ESM.docx]

Review History

**First round of review**

**Reviewer 1**

**Were you able to assess all statistics in the manuscript, including the appropriateness of statistical tests used?**

Yes, I'm able to access statistical analysis in the manuscript

**Were you able to directly test the methods?**

No

**Comments to author:**

In this work, Cheng and colleagues proposed a novel computational method, MTSplice, to predict the tissue specific genetic effects on alternative splicing. This method combines MMSplice, a tissue-independent genetic effect predictor, and TSplice, a tissue specific Psi predictor. This method has been extensively evaluated on GTEX data with 56 tissues and illustrated on a case study autism spectrum disorder (ASD). This topic is of high interest in genome biology and this type of method may have broad utilities and applications. The paper is generally well written, and the software looks under well development and maintenance.

1) It is logically sound to combine tissue free model (MMSplice, large scale) with tissue specific model (TSplice, smaller scale) to enhance to the prediction of tissue specific genetic effects on alternative splicing. However, by adding the TSplice module seems only decrease 1.5% on RMSE (0.140 with MTSplice to 0.138 MMSplice, Fig 6B), with the cost of adding additional complexity. More strikingly, the MTSplice gives much higher prediction error on Testis comparing to MMSplice (Fig 6B). This increased error is order of magnitude higher than the reduced error it offers in any other tissue, even though the RMSE is reduced in majority of tissues. This is worrying as the accuracy of TSplice itself is only moderate (median Spearman rho=0.25, Fig 5B, across 56 tissues), hence may introduce high variance on MTSplice's performance when comparing to MMSplice.

2) The measure of MTSplice is partially based measured value: Psi^{ref} in Eq (6) and Eq (10). This setting is practically reasonable; however, it seems unfair when comparing to MMSplice, which predict the Psi^{ref} - Psi^{alt} directly, hence may result in higher variance for subtraction over two unknown variables. It is not clear if this setting is makes MTSplice perform better than MMSplice, and how much. If possible, it is better to rule out.

3) When benchmarking TSplice in Fig 4 & 5, it is lack of comparison to other published methods, even though there are multiple well-developed ones, for example SPANR, and Xiong et al Bioinfo 2011 that were mentioned in introduction. Even linear regression models with commonly used sequence features (splice site motifs, conservation scores, etc) on a per tissue base (instead of multi-task setting) might be more informative than a simple average baseline used in here. In other words, the benchmarking of TSplice module can be more comprehensive.

4) line 44 on page 7: should this be MTSplice instead of MMSplice for Fig 6A? If so, it would be interesting to present the results on MMSplice too.

**Reviewer 2**

**Were you able to assess all statistics in the manuscript, including the appropriateness of statistical tests used?**

No

**Were you able to directly test the methods?**

No

**Comments to author:**

The authors present a new algorithm named MTSplice for the prediction of tissue-specific splicing changes resulting from genetic variants. MTSplice combines prediction from the authors' previous splicing predictor MMSplice and a new algorithm called TSplice. The combined system aims to combine the advantages of the individual components: MMSplice is designed to predict variant effects well as a result of being trained on millions of random sequences from MPRA experiments and TSplice, which is trained on tissue-specific PSI measurements from RNA-Seq from healthy human donors. The objective for the combined algorithm is for MMSplice to provide accurate predictions of variant effects and for TSplice to provide accurate predictions of tissue differences in splicing levels.

The work is of considerable interest to the fields of RNA splicing and transcriptomics as no splicing prediction tool currently exists that can be considered accurate in terms of predicting tissue-specific quantitative effects of genetic variants. A reliable and accurate predictor of tissue-specific variant effects on splicing levels would be of obvious importance to the community and would enable many downstream applications, including further study into tissue-specific splicing regulators and the identification and classification of disease-causing variants.

The MTSplice is distributed both as part of the "model zoo" Kipoi as well as a stand-alone Python package, with the source available from Github and the training data available from its original sources. This should be sufficient to make using the model for new predictions easy for other researchers, as well as making the analysis reproducible. The authors are requested to address the following comments in a revised manuscript.

Major:

1. While the authors present multiple pieces of evidence for the improved performance of MTSplice over MMSplice, additional evidence is needed to conclusively demonstrate the improvement and the sensitivity of MTSplice in predicting tissue-dependant splicing changes.

The authors mainly analyze performance differences between MMSplice and MTSplice in terms of root mean square error on different tissues. Overall, the study reports a statistically significant performance increase, but the improvement is small in terms of tissue-averaged variant prediction (RMSE of 0.140 vs 0.138) as well as tissue-specific prediction (Figure 6B). It is not clear if such an improvement would be of much practical significance. It is possible that averaging RMSE over all tissues dilutes the effect of improved predictions in a single tissue, i.e. a tissue-specific variant may change splicing in only one tissue and this improvement may not be visible when averaging across all tissues.

Achieving improvement in single tissues would serve the application better and would be more likely to demonstrate performance that has a practical impact. Accordingly, it is recommended that the authors perform additional validation of the model to assess tissue-specific improvements with MTSplice. For example, they should determine how reliably MTSplice can identify which tissue-dependent splicing patterns are affected by variants and whether the predicted changes are in the right direction. Does the method accurately predict neural-specific effects of specific variants, and not just small splicing changes with high precision?

2. An important detail is that TSplice is not designed to predict the absolute splicing level in each tissue, but the logit-difference in splicing level from the mean Δlogit(Ψ_(e,t) ) instead. This requires the average tissue splicing level (Ψ_e ) ̅ to compute the actual tissue-specific splicing level Ψ_(e,t). The manuscript does not go into detail on how to obtain this value, but we assume that it must be quantified from RNA-Seq data. If this is the case, then the authors should provide more detail to point out this choice and clearly state the advantages and disadvantages of this approach: e.g. are the authors distributing the average splicing levels of all annotated exons alongside the algorithm such that users can compute absolute PSI levels when required? Do users who want to predict the splicing levels of novel cassette exons need to compute this quantity from RNA-Seq data themselves?

3. In their analysis of ASD-associated variants, the authors select for variants that have a change of at least 0.05 in MMSplice. Rather than limiting the analysis to variants that are already classified to alter splicing by MMSplice, it is important to know if MTSplice can identify neural-specific variants that are being missed by MMSplice, which would provide significant evidence for the authors' claim that MTSplice can be used to predict the tissue-specific effects of potentially disease-causing mutations.

4. The paper provides a rather superficial analysis of the motifs that are learned by the convolutional neural network and mentions that the model detects instances of PTBP1/2, MBNL1 and NOVA1/2 binding sites. A deeper analysis of the features and motifs detected by the model, especially evidence that the model can detect known tissue-specific splicing elements and that detection of these motifs is driving tissue-specific predictions, would substantially strengthen the analysis. Furthermore, what would greatly enhance the manuscript is if the authors could use their model to predict the role of new and unexpected motifs (e.g. in less well studied tissues) and validate these experimentally.

Minor:

1. The authors should provide details on how the sequence motifs in Figure S4 were computed in the Methods section.

2. Figure 1B: This figure would be easier to read if it was rotated. It would also be helpful to add hierarchical clustering to the tissues.

3. Figure 6A: The caption states that tissue-specific variants are plotted in cyan and others in orange, but the figure legend shows the opposite.

4. Figure S4: The known binding motifs/PWMs for these RBPs should be shown below these motifs and a statistical test should be used to confirm the match (e.g. TOMTOM package).

**Authors Response**

**Point-by-point responses to the reviewers’ comments:**

Reviewer reports:

Reviewer #1: In this work, Cheng and colleagues proposed a novel computational method, MTSplice, to predict the tissue specific genetic effects on alternative splicing. This method combines MMSplice, a tissue-independent genetic effect predictor, and TSplice, a tissue specific Psi predictor. This method has been extensively evaluated on GTEX data with 56 tissues and illustrated on a case study autism spectrum disorder (ASD). This topic is of high interest in genome biology and this type of method may have broad utilities and applications. The paper is generally well written, and the software looks under well development and maintenance.

1) It is logically sound to combine tissue free model (MMSplice, large scale) with tissue specific model (TSplice, smaller scale) to enhance to the prediction of tissue specific genetic effects on alternative splicing. However, by adding the TSplice module seems only decrease 1.5% on RMSE (0.140 with MTSplice to 0.138 MMSplice, Fig 6B), with the cost of adding additional complexity. More strikingly, the MTSplice gives much higher prediction error on Testis comparing to MMSplice (Fig 6B). This increased error is order of magnitude higher than the reduced error it offers in any other tissue, even though the RMSE is reduced in majority of tissues. This is worrying as the accuracy of TSplice itself is only moderate (median Spearman rho=0.25, Fig 5B, across 56 tissues), hence may introduce high variance on MTSplice's performance when comparing to MMSplice.

*Response:*

*We thank the reviewer for underscoring the relevance of predicting tissue-specific splicing alterations and appreciating the maturity of our software. Despite its biomedical relevance, predicting the tissue-specific effects of splicing variants is an under-explored area. One of the major hurdles is the lack of extensive data of variants with tissue-specific splicing effects. Most variants observed so far (e.g. GTEx) have no or very small tissue-specific effects, even though they might have a strong effect on every tissue (e.g. splice site mutations). This has a direct implication regarding the assessment of the performance improvement of MTSplice compared to non-tissue specific models..*

*Our revised manuscript addresses this concern in a four-fold manner:*

*i) Following a suggestion from reviewer #2, we have now assessed the performance restricted to tissue-specific differential percent spliced-in. To this end, we considered data points for which delta-Psi deviates by more than 0.2 from its average across tissues (Supplementary Figure S6B). The RMSE difference compared to MMSplice improves further on these data. It remains however modest, especially for non-brain tissues. One should note that the RMSE might also be hard to improve because the sample size (number of individual homozygous) is often low and the effect sizes are typically small.*

*ii) We now comment on the lack of performance for testis in the discussion. Testis shows up as an outlier. The improvements across all other tissues are consistent. We do not think that the poorer performance in testis indicates a lack of robustness. Instead, we think that MTSplice fails on testis because of its unique transcriptional biology (Xia et al., Cell, 2020, https://doi.org/10.1016/j.cell.2019.12.015), which may affect splicing in a way that the model could not capture.*

*iii) In response to Reviewer #2 question 3. We now show that MTSplice can capture tissue-specific disease signals that would otherwise be missed by tissue-agnostic MMSplice.*

*iv) Our contribution is also valuable for the computational biology community. We have built MTSplice in a modular way. Unlike earlier tissue-specific attempts we provide code for others to build upon. Colleagues, who could, for instance, generate cell-type-specific MPRA data will be able to build upon our model by substituting the TSplice module with an improved one (Eq. 10). We emphasize this further in the discussion.*

R1: 2) The measure of MTSplice is partially based measured value: Psi^{ref} in Eq (6) and Eq (10). This setting is practically reasonable; however, it seems unfair when comparing to MMSplice, which predict the Psi^{ref} - Psi^{alt} directly, hence may result in higher variance for subtraction over two unknown variables. It is not clear if this setting is makes MTSplice perform better than MMSplice, and how much. If possible, it is better to rule out.

*Response: Actually, MMSplice does not predict Psi^{alt} - Psi^{ref} but logit(Psi^{alt}) - logit(Psi^{ref})). Therefore, MMSplice also needs Psi^{ref} to perform Psi^{alt} - Psi^{ref} predictions. We now clarify that the two methods are compared on an equal footing by explicitly providing the pendant of the MTSplice prediction model (equation 10) for the MMSplice prediction model (new Equation 11).*

R1: 3) When benchmarking TSplice in Fig 4 & 5, it is lack of comparison to other published methods, even though there are multiple well-developed ones, for example SPANR, and Xiong et al Bioinfo 2011 that were mentioned in introduction. Even linear regression models with commonly used sequence features (splice site motifs, conservation scores, etc) on a per tissue base (instead of multi-task setting) might be more informative than a simple average baseline used in here. In other words, the benchmarking of TSplice module can be more comprehensive.

*Response: Even though SPANR was trained to predict tissue-specific effects for 16 human tissues, its tissue-specific performance was not reported. Moreover, the tissue-specific predictions were not made available publicly. The public version of SPANR, available at http://tools.genes.toronto.edu/, specifies that “The tool reports the maximum mutation-induced change in PSI across 16 tissues”.*

*The method of Xiong et al Bioinfo 2011 was developed for Mouse. We also did not find the model to be publically available.*

*To provide a more comprehensive benchmark, we followed the suggestion of the reviewer and investigated more realistic baseline models.*

*To this end, we have now trained tissue-specific L2-regularized linear models using relevant sequence features. These features are the binding motifs of well-characterized tissue-specific spacing factors NOVA1/2, MBNL1, PTBP1/2, RBFOX1, as well as the 2,272 significant hexamers reported by Ke et al., Genome Res., 2011. We additionally also used splice site contexts, considering the sequence region of MaxEntScan: 20nt intron, 3nt exon from the acceptor side, 6nt intron, 3nt exon from the donor side. The regularization parameter of this baseline model was selected using the same cross-validation scheme as TSplice.*

*Even though the performance of TSplice and the baseline correlate across tissues (R=0.373), TSplice outperforms the baseline model in all 56 tissues (updated Fig S2A). When evaluated per exon, TSplice shows higher Spearman correlation for 63.2% of the exons compared to the ridge regression baseline (updated Fig S2B).*

R1: 4) line 44 on page 7: should this be MTSplice instead of MMSplice for Fig 6A? If so, it would be interesting to present the results on MMSplice too.

*Response: Indeed a side-by-side comparison of MTSplice with MMSplice on a representative tissue is interesting. Therefore, we now present scatterplots for MMSplice in 6A and for MTSplice in the new 6B.*

Reviewer #2: The authors present a new algorithm named MTSplice for the prediction of tissue-specific splicing changes resulting from genetic variants. MTSplice combines prediction from the authors' previous splicing predictor MMSplice and a new algorithm called TSplice. The combined system aims to combine the advantages of the individual components: MMSplice is designed to predict variant effects well as a result of being trained on millions of random sequences from MPRA experiments and TSplice, which is trained on tissue-specific PSI measurements from RNA-Seq from healthy human donors. The objective for the combined algorithm is for MMSplice to provide accurate predictions of variant effects and for TSplice to provide accurate predictions of tissue differences in splicing levels.

The work is of considerable interest to the fields of RNA splicing and transcriptomics as no splicing prediction tool currently exists that can be considered accurate in terms of predicting tissue-specific quantitative effects of genetic variants. A reliable and accurate predictor of tissue-specific variant effects on splicing levels would be of obvious importance to the community and would enable many downstream applications, including further study into tissue-specific splicing regulators and the identification and classification of disease-causing variants.

The MTSplice is distributed both as part of the "model zoo" Kipoi as well as a stand-alone Python package, with the source available from Github and the training data available from its original sources. This should be sufficient to make using the model for new predictions easy for other researchers, as well as making the analysis reproducible. The authors are requested to address the following comments in a revised manuscript.

Major:

1. While the authors present multiple pieces of evidence for the improved performance of MTSplice over MMSplice, additional evidence is needed to conclusively demonstrate the improvement and the sensitivity of MTSplice in predicting tissue-dependant splicing changes.

The authors mainly analyze performance differences between MMSplice and MTSplice in terms of root mean square error on different tissues. Overall, the study reports a statistically significant performance increase, but the improvement is small in terms of tissue-averaged variant prediction (RMSE of 0.140 vs 0.138) as well as tissue-specific prediction (Figure 6B). It is not clear if such an improvement would be of much practical significance. It is possible that averaging RMSE over all tissues dilutes the effect of improved predictions in a single tissue, i.e. a tissue-specific variant may change splicing in only one tissue and this improvement may not be visible when averaging across all tissues.

Achieving improvement in single tissues would serve the application better and would be more likely to demonstrate performance that has a practical impact. Accordingly, it is recommended that the authors perform additional validation of the model to assess tissue-specific improvements with MTSplice. For example, they should determine how reliably MTSplice can identify which tissue-dependent splicing patterns are affected by variants and whether the predicted changes are in the right direction. Does the method accurately predict neural-specific effects of specific variants, and not just small splicing changes with high precision?

*Response: On top of the detailed answer to the point 1 of reviewer #1, we would like to address the reviewer’s concern that averaging over tissues dilutes the effect of improved predictions in a single tissue, and that whether MTSplice is accurate in certain (neural) tissues.*

*To address the reviewer’s concern about the diluted effect, we further compared MTSplice and MMSplice with variants that have large tissue-specific effects (|delta-psi - delta-psi_avg| > 0.2) and only in tissues where these tissue-specific effects are shown. We again evaluated tissues with at least 10 valid variants. The improvement of MTSplice is more prominent in this setting (new Supplementary Figure S6). We would like to stress that the RMSE is computing using signed predictions and thus the direction of change is taken into account. Moreover, we are not averaging RMSE per variant across tissues but per tissue across variants. See also the response to reviewer #1 point 1).*

*Furthermore, we consistently see a higher performance on brain tissues compared to non-brain tissues in terms of TSplice performance (Figure 4C) and variant effect prediction (new Supplementary Figure S6).*

*The improvements of MTSplice over MMSplice measured by RMSE on those tissue-specific differential Psi remain modest. The variants in the benchmark are naturally occurring variations in GTEx with mostly small effect size. Given the reasonable predictions that TSplice does (Figures 4-5), we may suffer from an absence of evidence when evaluating the added value on tissue-specific variant effect prediction.*

R2: 2. An important detail is that TSplice is not designed to predict the absolute splicing level in each tissue, but the logit-difference in splicing level from the mean Δlogit(Ψ_(e,t) ) instead. This requires the average tissue splicing level (Ψ_e ) ̅ to compute the actual tissue-specific splicing level Ψ_(e,t). The manuscript does not go into detail on how to obtain this value, but we assume that it must be quantified from RNA-Seq data. If this is the case, then the authors should provide more detail to point out this choice and clearly state the advantages and disadvantages of this approach: e.g. are the authors distributing the average splicing levels of all annotated exons alongside the algorithm such that users can compute absolute PSI levels when required? Do users who want to predict the splicing levels of novel cassette exons need to compute this quantity from RNA-Seq data themselves?

*Response: We provide tissue-specific Psi levels along with the package for cassette exons in ASCOT. ASCOT uses the de novo method to compute PSI for exons (Ling et al. 2020), therefore it also contains expressed exons absent from standard annotations.*

*For novel cassette exons, users indeed need to compute PSI from RNA-Seq data themselves. We now explain this point in the Availability section, and also added example code in the github repository.*

R2: 3. In their analysis of ASD-associated variants, the authors select for variants that have a change of at least 0.05 in MMSplice. Rather than limiting the analysis to variants that are already classified to alter splicing by MMSplice, it is important to know if MTSplice can identify neural-specific variants that are being missed by MMSplice, which would provide significant evidence for the authors' claim that MTSplice can be used to predict the tissue-specific effects of potentially disease-causing mutations.

*Response: We agree with the reviewer that it is important to know whether MTSplice can capture tissue-specific mutational signals related to ASD that would otherwise be missed by non-tissue specific MMSplice. To answer this question, we analyzed further variants with abs(MMSplice) < 0.05. As expected, the delta-logit-Psi scores by MMSplice do not show significant differences between the Proband group and the healthy Sibling group. However, for those variants with abs(MMSplice) < 0.05 yet abs(MTSplice) > 0.05, this difference is significant in the Frontal Cortex tissue (new Supplementary Figure S8A). Overall, the predicted differences between Proband and Sibling groups are significantly larger in neural tissues (new Supplementary Figure S8B). This result shows that MTSplice indeed captures relevant tissue-specific variants missed by MMSplice.*

R2: 4. The paper provides a rather superficial analysis of the motifs that are learned by the convolutional neural network and mentions that the model detects instances of PTBP1/2, MBNL1 and NOVA1/2 binding sites. A deeper analysis of the features and motifs detected by the model, especially evidence that the model can detect known tissue-specific splicing elements and that detection of these motifs is driving tissue-specific predictions, would substantially strengthen the analysis. Furthermore, what would greatly enhance the manuscript is if the authors could use their model to predict the role of new and unexpected motifs (e.g. in less well studied tissues) and validate these experimentally.

*Response: We had attempted to discover new motifs using model interpretation tools but did not discover motifs that we would confidently report or take up for functional validation. This failure may be because de novo motif discovery from deep neural network is less established for RNA-based model than for DNA-based models. Moreover, TSplice is modelling a complex process, splicing, which involves many factors. This in contrast to models predicting targeted binding assays (CLIP and alike) that are determined by specific factors. Therefore, we instead asked what effects TSplice associates with sequence motifs of well-characterized tissue-specific splicing regulators: NOVA1/2 (TCAT), PTBP1/2 (CTTTTC), MBNL1 (GCTTGC). To this end, we have generated in silico mutated sequences by injecting the sequence motifs of these factors at each various positions along 1,000 randomly selected sequences of the test set. We then computed the TSplice score difference between each mutated sequence and its original counterpart.*

*TSplice predicts that cassette exons with a NOVA1/2 motif in the upstream intron are less spliced-in (i.e. more skipped) in the brain (blue, negative values) compared to other tissues (red, positive values) (new Supplementary Figure S5). Since the RNA-binding protein NOVA1 is neuron-specifically expressed, these TSplice predictions are consistent with a NOVA1 repressive role when binding in the upstream intron [Ule et al., 2006 Nature].*

*Moreover, exons with an MBNL1 motif in the upstream intron are predicted to be more spliced-in in the brain and muscle than in other tissues (new Supplementary Figure S5). This is consistent with prior knowledge that MBNL1 suppresses splicing when binding to upstream introns and that MBNL1 is less expressed in the brain and muscle than in other tissues [Wang et al., 2012 Cell].*

*The interpretation of the effects of PTBP1/2 is more complex since it is recognized by two competing factors with the opposite pattern of expression [Linares et al., eLife, 2015 https://elifesciences.org/articles/09268].*

*We now added this analysis to the manuscript.*

R2: Minor:

1. The authors should provide details on how the sequence motifs in Figure S4 were computed in the Methods section.

*Response: We added the method description in the Methods section.*

R2: 2. Figure 1B: This figure would be easier to read if it was rotated. It would also be helpful to add hierarchical clustering to the tissues.

*Response: We rotated the heatmap and also added hierarchical clustering to tissues.*

R2: 3. Figure 6A: The caption states that tissue-specific variants are plotted in cyan and others in orange, but the figure legend shows the opposite.

*Response: We now fixed this.*

R2: 4. Figure S4: The known binding motifs/PWMs for these RBPs should be shown below these motifs and a statistical test should be used to confirm the match (e.g. TOMTOM package).

*Response: We are now reporting statistical assessments using TOMTOM on the RNA motif database RNACompete (Ray et al. 2013). P-values are shown on the plot. The NOVA1/2 motif is not present in RNACompete, therefore no P-value is shown. Motif search using the ATtRACT database was used to annotate this motif instead.*

**Second round of review**

**Reviewer 1**

Most of my concerns have been addressed. Besides potential capability of capturing tissue-specific disease signals, the author discussed the limited prevalence and modest level of tissue-specific genetic effects on splicing. Nevertheless, the open-source, well-modular methods, including both TSplice and MTSplice, are valuable to the computational biology community.

**Reviewer 2**

The authors have diligently addressed our comments and we are pleased to recommend publication in Genome Biology. As a minor note, it is suggested that the authors update the review references in their Introduction as several excellent reviews have been published on the topic of alternative splicing in the past few years.
